# The State of the Art of Theranostic Nanomaterials for Lung, Breast, and Prostate Cancers

**DOI:** 10.3390/nano11102579

**Published:** 2021-09-30

**Authors:** Lucas F. Freitas, Aryel H. Ferreira, Velaphi C. Thipe, Gustavo H. C. Varca, Caroline S. A. Lima, Jorge G. S. Batista, Fabiane N. Riello, Kamila Nogueira, Cassia P. C. Cruz, Giovanna O. A. Mendes, Adriana S. Rodrigues, Thayna S. Sousa, Victoria M. Alves, Ademar B. Lugão

**Affiliations:** 1 Nuclear and Energy Research Institute, IPEN-CNEN/SP, Sao Paulo 05508-000, Brazil; aryelhf@gmail.com (A.H.F.); vctqnf@mail.missouri.edu (V.C.T.); caroline.lima@usp.br (C.S.A.L.); jorgegabriel@alumni.usp.br (J.G.S.B.); fabiriello@yahoo.com.br (F.N.R.); kmnogueira@usp.br (K.N.); cassia.pris.cruz@gmail.com (C.P.C.C.); giovanna.asenjo@unifesp.br (G.O.A.M.); a9souz@outlook.com (A.S.R.); thayna.s.souza01@gmail.com (T.S.S.); vmalves@unifesp.br (V.M.A.); ablugao@gmail.com (A.B.L.); 2 MackGraphe-Graphene and Nanomaterial Research Center, Mackenzie Presbyterian University, Sao Paulo 01302-907, Brazil

**Keywords:** theranostics, nanomaterials, nanotechnology, lung cancer, breast cancer, prostate cancer

## Abstract

The synthesis and engineering of nanomaterials offer more robust systems for the treatment of cancer, with technologies that combine therapy with imaging diagnostic tools in the so-called nanotheranostics. Among the most studied systems, there are quantum dots, liposomes, polymeric nanoparticles, inorganic nanoparticles, magnetic nanoparticles, dendrimers, and gold nanoparticles. Most of the advantages of nanomaterials over the classic anticancer therapies come from their optimal size, which prevents the elimination by the kidneys and enhances their permeation in the tumor due to the abnormal blood vessels present in cancer tissues. Furthermore, the drug delivery and the contrast efficiency for imaging are enhanced, especially due to the increased surface area and the selective accumulation in the desired tissues. This property leads to the reduced drug dose necessary to exert the desired effect and for a longer action within the tumor. Finally, they are made so that there is no degradation into toxic byproducts and have a lower immune response triggering. In this article, we intend to review and discuss the state-of-the-art regarding the use of nanomaterials as therapeutic and diagnostic tools for lung, breast, and prostate cancer, as they are among the most prevalent worldwide.

## 1. Introduction

The unsolved oncologic challenges of current treatment regimens are hindered by (i) their inability to detect distant micrometastases and prognostic tumor aggressiveness, (ii) non-specific and non-selective delivery with poor biodistribution yielding toxicity, (iii) differentiation between indolent tumors and tumors exhibiting metastatic potential, and (iv) real-time monitoring and predictive treatment response—which affords physicians to adjust dosimetry to prevent overtreatment resulting in harmful side-effects or undertreatment that can lead to incomplete cancer remission. It is paramount to circumvent systemic distribution or off-target accumulation of therapeutics that can be detrimental to patients.

According to recent data, breast cancer is the most common cancer among women and is characterized by multifactorial pathogenesis [1]. There are three main types of breast cancer, classified according to biochemical and molecular characteristics, disease stage, and response to treatment: HER2-positive cancers (human epidermal growth factor receptor 2), the hormone receptor-positive cancers, and the triple-negative cancers (they are negative for HER2 receptors, estrogen receptors, and progesterone receptors). Their incidence depends mostly on ethnicity, age, and life habits, and both the therapeutic approach and the prognosis are dependent on the cancer types as well [2].

Prostate cancer, on the other hand, is the second most prevalent malignancy among men, and the prognosis is worse according to age, ethnicity, daily habits, and the time after diagnosis. The most common biomarker related to prostate cancer is PSA (Prostate-Specific Antigen), which is often elevated in prostate malignancies [3]. It can also be used as a targeting agent, according to recent data and some recent clinical trials. Although current treatment interventions, e.g., enzalutamide and other specific inhibitors or immunotherapeutics, have demonstrated modest progression-free survival increases, researchers keep searching for novel and more efficient approaches for prostate malignancies [4].

Finally, lung cancer is the lead cause of death worldwide due to malignant disease in both sexes. In spite of all the advances obtained throughout the last decades, the overall survival still remains low; therefore, the research in this field stays at a fast pace. The main risk factors for lung malignancies are smoking and air pollution, but it is known that there are genetic factors involved [5].

Advances in the synthesis and engineering of nanomaterials offer more robust and specific systems for the treatment of severe diseases, such as cancer, with technologies that combine therapy with imaging diagnostic tools in the so-called nanotheranostics [6]. The broad and most used definition of nanomaterials is provided by the regulatory organ International Organization for Standardization (ISO), which defines a nanomaterial as any material with external dimensions, internal structure, or surface structure within the nanometric scale (10^−9^ m), with an average size between 1 and 100 nm [7].

Synthetic nanomaterials are still the most commonly used compared to natural ones, either produced by chemical or physical methods, as those methods provide easy scalability and precise control on size and morphology. Another interesting factor is the possibility of anchoring or conjugating different reagents and chemical compounds with accuracy. One of the main concerns about synthetic nanomaterials, though, is the environmental impact they might have, which can be even higher than their bulk counterparts [8].

Most of the advantages of nanomaterials over the classic anticancer therapies come from their optimal size, which prevents the elimination by the kidneys and enhances their permeation in the tumor due to the abnormal blood vessels present in cancer tissues. Furthermore, the drug delivery and the contrast efficiency for imaging are enhanced, especially due to the increased surface area and the selective accumulation in the desired tissues. This property leads to the reduced drug dose necessary to exert the desired effect and to longer action within the tumor. Finally, they are made so that there is no degradation into toxic byproducts and lower immune response triggering [6].

In this article, we intend to review and discuss the state-of-the-art regarding the use of nanomaterials as therapeutic and diagnostic tools for lung, breast, and prostate cancer, as they are among the most prevalent worldwide.

## 2. Theranostic Nanomaterials

Nanotheranostics (the field of science focused on developing materials and devices that act both as therapeutic and diagnostic agents) represents one of the scientific frontiers involving different multidisciplinary aspects and can contribute to human health advancement. Nanotheranostic systems offer advantages when compared to conventional therapy, and diagnostic, pharmaceutical approaches applied separately [9]. Ideally, nanotheranostic systems should provide the therapeutic and diagnostic agent delivery exclusively to the desired location, minimizing the amount needed to obtain the desired effect and, consequently, adverse effects [10]. This specific targeting is achieved according to the design and materials used in its construction.

**Figure 1:**
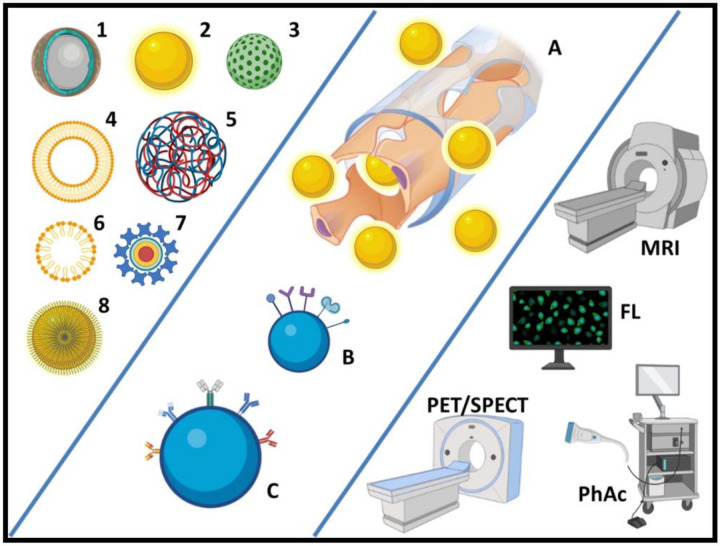
Most common nanoparticles for theranostic purposes in breast, lung, and prostate cancer: (1) core-shell NPs, (2) metal NPs for Magnetic Resonance Imaging (MRI) contrast, (3) mesoporous NPs, (4) liposomes, (5) polymer nanocapsules, (6) micelles, (7) quantum dots, (8) functionalized NPs; strategies for specific targeting: (A) enhanced permeation and retention effect, (B) functionalization with specific ligands, (C) functionalization with monoclonal antibodies; and main diagnostic applications of NPs for those cancers: (MRI) Magnetic Resonance Imaging, (FL) Fluorescence Imaging, (PET/SPECT) Positron-Emission Tomography/Single Photon-Emission Computer Tomography, (PhAc) Photoacoustic Imaging.

In this field, a wide variety of component systems that modulate the speed and direct the drug to be released at a specific site have been the nanotechnology research subject [11]. Among the most studied systems, there are quantum dots, liposomes, polymeric nanoparticles, inorganic nanoparticles, magnetic nanoparticles, dendrimers, and gold nanoparticles (Figure 1) [12].

Quantum dots

Quantum dots (QD) are light-emitting inorganic nanocrystals composed of semiconductor elements from the periodic groups II–VI or III–V [13]. They have unique optical characteristics, absorbing the light energy intensely in the spectral region from the ultraviolet to the near-infrared (NIR) and emitting fluorescence by narrow and symmetrical bands whose position depends on both the size of the nanocrystal and the type of semiconductor used [14]. The core materials are generally composed of CdSe, CdTe, CdS, ZnS, ZnSe, PbS and PbSe, InP, and InGaP, with a core-shell structure of 1 to 10 nm in diameter [15]. The inorganic coating with ZnS and CdS on the particle’s surface improves the photoluminescent quantum efficiency and protects the nucleus from oxidation in biological environments, as they are formed from inorganic elements whose cytotoxicity can be significant even at low concentrations [16,17].

These systems can release toxic ionized metals exposed to oxidation promoted by the environment. However, QD’s degradation and cytotoxicity can be minimized by encapsulating it with a biocompatible system [18]. The use of specific compounds, such as aptamers to functionalize QD can increase their affinity with selected locations in the organism, enabling its selective transport or drug delivery [19]. QDs are considered an alternative to molecular fluorophores, such as fluorescein and rhodamine, as they have better photostability and long-lasting fluorescence time (~10–100 ns), enabling their use for an extended period of time without losing efficiency, even in extremely low concentrations [20].

Polymeric Nanoparticles

Polymeric nanoparticles have a diameter of less than 1 µm and can be prepared from natural or synthetic polymers. They include nanocapsules and nanospheres that differ in composition and structural organization [21]. In general, polymer-based nanotheranostic materials comprise: (i) a polymer component that offers stabilization and biocompatibility, (ii) a therapeutic agent (i.e., small molecule drug, siRNA, etc.), and (iii) an imaging agent (i.e., MRI contrast agent, radionuclide, fluorophore, etc.) [22]. They are considered one of the most promising tools for theranostics applications.

The nanocapsules are constructed with a polymeric shell that surrounds a hydrophobic core. The drug is usually dissolved in the core but can also be present in the nanocapsule’s surface [23]. On the other hand, nanospheres are formed by a polymeric matrix, and a drug can be retained or adsorbed on the matrix. Synthetic polymers, such as poly (lactic-co-glycolic acid) (PLGA), Poly lactic Acid (PLA), and natural polymers, such as albumin, collagen, and chitosan, have been efficiently used in these systems [24].

Polymer-based distribution systems are preferred because they have an inherent versatility in their structures. The potential for modifying their surface (with peptide molecules, proteins, etc.) allows specific targeting of therapeutic and/or diagnostic agents to specific regions, increasing the effectiveness, sensitivity, and specificity of the therapy and diagnosis approach. Furthermore, they can be easily adapted to improve the theranostic conjugate solubility and biocompatibility. [25].

Liposomes

Liposomes are concentric vesicles with one or more lipid bilayers of natural or synthetic phospholipids and cholesterol. [26]. Liposomes are used for drug delivery and as diagnostic agents due to several advantages: they are suitable for transport compounds with different physicochemical characteristics (hydrophilic, hydrophobic, and amphiphilic); they can be functionalized in order to decrease toxicity and adverse effects; they provide protection and a controlled release of the diagnostic agent in the body; they are considered suitable for different routes of administration and a wide variety of physiological conditions; their size can be tuned according to necessity; and, finally, due to their nature, liposomes are generally biocompatible [27]. Despite some disadvantages in using liposomes that might appear in certain conditions, i.e., their high production costs, low stability (phospholipids are easily hydrolyzed and oxidized) associated with a reduced half-life, liposomes are one of the main nanotechnological resources when it comes to encapsulating drugs and active compounds, and many clinical trials are currently using this approach.

Micelles

Micelles, with a size ranging between 5 and 100 nm, are colloidal dispersions of amphiphilic substances, with hydrophilic and hydrophobic regions, which self-organize in an aqueous solution through the establishment of hydrophobic interactions [28]. Micelles have been widely studied as drug carriers, being prepared from various amphiphilic materials, allowing the increase of hydrophobic molecules solubility and the incorporation of multiple functionalities in a single structure [12].

Usually, micelles are created through the self-assembly of copolymers in an aqueous medium that occurs in a concentration-dependent manner [29]. The drug and the diagnostic agents can be attached to the polymer before forming the micelle or become trapped in the micelle’s hydrophobic core. The micellar structure is highly advantageous in drug administration since the hydrophobic core increases the solubility of hydrophobic drugs and the hydrophilic periphery protects them. Micelles are not easily recognized by phagocytes, enabling the drug to remain in the bloodstream until it reaches the therapeutic target. Additionally, they can be composed of biocompatible and biodegradable copolymers and are quickly eliminated from the body by renal excretion, which contributes to their low immunogenicity [30].

Nevertheless, some hindrances must be eliminated in order for micelles to be used in actual therapeutic interventions. For instance, their stability in the bloodstream must be improved so that the micelle concentration in the blood remains sufficiently high, and the encapsulation efficiency might also be improved with some strategies, such as pi-stacking interactions, free-radical polymerization, and entrapment of micelles into other structures, i.e., CaCO_3_ crystals [28].

Dendrimers

The term dendrimer refers to their characteristic appearance, which are monodispersed macromolecules with a highly branched and regular three-dimensional structure around the nucleus [31]. Two strategies may be used to synthesize them: the dendrimer can grow out of a central nucleus in a process known as a divergent method, or prepared by the convergent method, in which the dendrimer is synthesized from the periphery to the interior, ending in the nucleus [32].

They present some ideal characteristics for a release system: the possibility of structural control of size and shape; biocompatibility and low toxicity; high capacity for incorporating substances in their interior or on the surface; the ability to promote controlled drug release; the different functional structures on its surface are subject to modification for labeling with cell-specific molecules; low immunogenicity; cell adhesion, endocytosis, and appropriate cell trafficking depending on their surface clusters; ability to promote drug isolation at the molecular level during transit to target cells and thereby to protect it from inactivation; and, high solubility in a large number of organic solvents favoring the process and a rapid dissolution [33,34]. The main concern about dendrimers, however, is regarding their cytotoxicity depending on the polymer they are made of. In general, some structural modifications must be made, or more biocompatible polymers must be recruited in order to offer more biologically suited alternatives [35].

Gold-based nanotheranostic agents

The theranostic inorganic nanoparticle application using gold (AuNPs) has been investigated due to the unique combination of their intrinsic optical and thermal properties. The AuNPs have adjustable size, shape, and surface chemistry, with morphology varying from spheres, cubes, rods, clusters to threads, requiring precise shape control, which influences physical properties and affects their use in theranostic applications [36]. Due to the strong interaction between the thiol groups and gold, the modification of the AuNPs’ surface is predominantly driven by the addition of thiolated species. The functionalization of gold nanoparticles can be used to orient and release drugs to specific cell sites or groups [37].

One of the most important properties of AuNPs comes from the interaction with an electromagnetic field, which, at specific frequencies, can induce a resonant and coherent oscillation of the free electrons on the surface of the nanostructures. This oscillation is known as surface plasmon resonance (SPR) [38,39]. Gold nanoparticles can absorb this energy and convert it into heat, which causes tumor cell ablation, mainly due to the destruction of cell membranes [40,41].

Another theranostic approach is the use of radioactive gold nanoparticles (^198^AuNP and ^199^AuNP). Both radionuclides are beta (−β) and gamma (γ) emitters, where beta particles are used to destroy the tumor tissue and gamma rays to scintigraphy or single photon emission computed tomography/computed tomography (SPECT/CT) image acquisition [42].

Magnetic Nanoparticles

Magnetic nanoparticles (MNPs) have been considered important nanomaterials in theranostic nanoparticle design, especially iron oxide nanoparticles (IONPs) composed of magnetite (Fe_3_O_4_) or hematite (Fe_2_O_3_) [43]. They are used for several purposes, such as contrast to magnetic resonance imaging (MRI), drug delivery, controlled/sustained release, and hyperthermia treatment. Some of them are in clinical trials for humans [44].

MNPS in theranostic approaches has important advantages: IONPs are paramagnetic and can be used as imaging agents to diagnose and monitor pathological conditions and release drugs by applying an external magnetic field in target tissue [45]. These systems have simultaneous therapeutic and diagnostic functions in oncology due to their potential for hyperthermia generation (42−45 °C) after an alternating external magnetic field application, with consequent cell death or making tumor tissue more sensitive to the radiation effects and some antineoplastic drugs. Cell death occurs selectively (in the tissues that contain nanoparticles), leading to reduced adverse effects. In addition to the advantages mentioned above, it is essential to highlight that these nanoparticles used are considered safe as they are easily degraded and metabolized in the serum iron pool to form hemoglobin or other metabolic processes [46,47].

The following tables (Table 1 and Table 2) summarize some relevant studies using nanomaterials for breast, lung, and prostate cancer and their synthesis methods, as well as some clinical trials that are currently using nanoparticles for several purposes. Furthermore, Figure 1 summarizes the main nanomaterials as well as their possible applications addressed in this review.

## 3. Targeting Strategies

Tumor Internationalization and Tumor Microenvironments: Active and Passive Targeting

The delivery of theranostic materials can be facilitated through active and passive targeting. Passive targeting is based on two physiological processes, which include the convection process driven by rhythmic blood circulation pressure responsible for the transport of large molecules through the tumor microvasculature, and diffusion process facilitated by a concentration gradient and mainly responsible for the transport of highly lipophilic and low molecular weight compounds across the cell membrane [75]. On the other hand, active targeting is achieved by specific interaction between the target cell and the carrier by selective cognate binding efficiency to overexpressed receptors in the tumor site, thereby improving cell recognition and uptake [76]. Active targeting with conjugated ligands avoids the destruction of surrounding nearby healthy cells/tissues. However, it is imperative to consider that the carriers must reach the tumor, thereafter, internalize within the tumor cells, and this is achieved by the enhanced permeability and retention (EPR) effect through proper cellular trafficking throughout the body and accumulation in the tumor [75,77].

**Figure 2:**
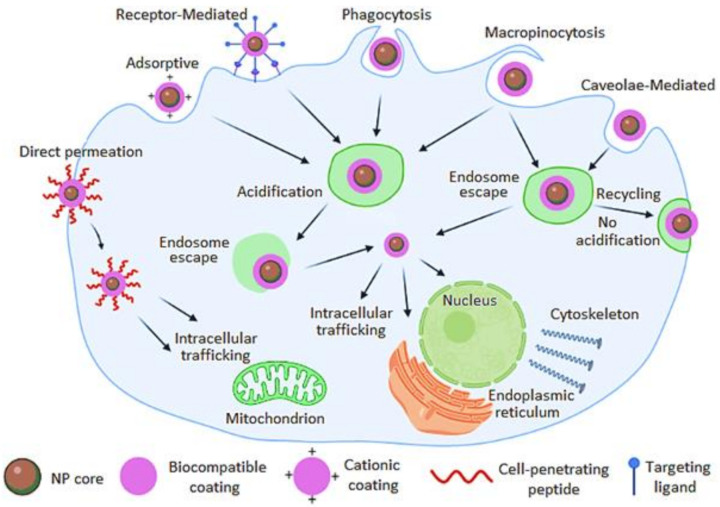
Passive and active targeting of theranostic nanomaterials for cellular internalization in tumors via direct permeation and various endocytosis mechanisms.

Most nanotheranostic agents are internalized through endocytosis pathways, which are facilitated by receptor-mediated endocytosis and adsorptive endocytosis (i.e., clathrin-coated pits) [78]. Cellular internalization can also occur via phagocytosis, which is the main uptake mechanism into macrophage cells, while caveolae-mediated endocytosis occurs in non-clathrin-coated plasma membrane present on the surface of some cells. Micropinocytosis is a fluid-phase endocytosis mechanism and other mechanisms that do not involve clathrin or caveolae. Receptor-mediated endocytosis is achieved by attaching a targeting moiety/ligand to the surface of the theranostic agent that recognizes overexpressed receptors on the surface of the tumor cell, as shown in Figure 2. Generally, when the theranostic agent is bound to the cell surface receptor, the theranostic agent is engulfed and once internalized, the theranostic agent is wrapped within an endocytic vesicle [78]. The theranostic must extravasate the endosome, proper cellular-trafficking to the intracellular site of action (cytoplasm, nucleus, mitochondria, Golgi apparatus, or cytoskeleton) to improve the therapeutic efficacy and exhibit real-time monitoring treatment response through imaging [79].

Targeting Moieties/Ligands

**Figure 3:**
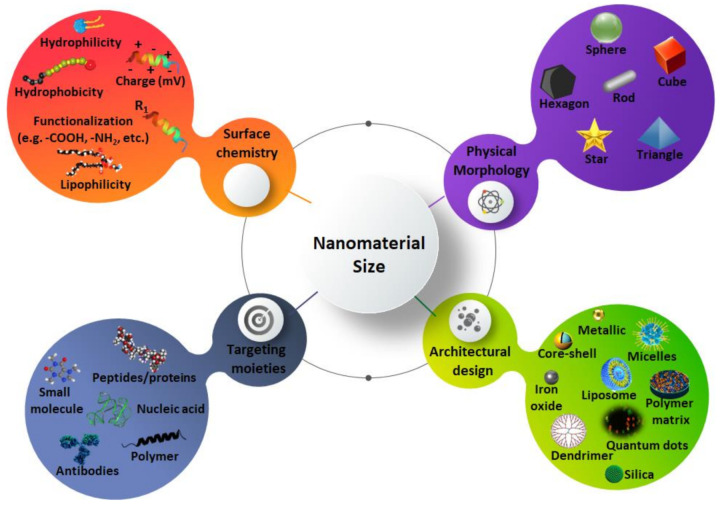
Overall theranostic architecture for theranostic delivery.

Lee and colleagues [80] emphasized the importance of new strategies to discover target antigens and combinatorial targeting of antigens to overcome the heterogeneity and plasticity inherent to solid tumors. The theranostic agent, once extravasated from the blood into the tumor site, it is imperative for the theranostic agent to achieve optimal tumor internalization to confer efficient therapeutic payload without affecting surrounding normal tissues [77]. There are several targeting strategies, according to the different materials used as targeting agents (Figure 3 and Table 3).

Antibodies

Antibodies (mAbs) are proteins with two epitope-binding sites with extraordinary selectivity and specificity to their antigen. The mAbs; such as humanized bevacizumab (Avastin), for VEGF targeting; humanized trastuzumab (Herceptin), which targets HER2; chimeric cetuximab (Erbitux), which targets the human epidermal growth factor receptor (EGFR); chimeric rituximab (Rituxan), for targeting hematopoietic differentiation antigen CD20; and humanized panitumumab (Vectibix), for targeting EGFR, can be conjugated to theranostic nanomaterials.

Aptamers

Produced using systemic evolution of ligands by exponential enrichment (SELEX), aptamers are small synthetic ligands (~15 kD) that have demonstrated superior binding affinity with high specificity when compared with antibodies (~150 kD). Aptamers can be conjugated to FeONPs via peptide bonds between the amine group of the aptamer and the carboxylic group of FeONPs.

Peptides

Peptides have a low molecular size, are highly stable, and exhibit high specificity and low immunogenicity [81]. They can promote selective intracellular delivery of a plethora of therapeutic agents, and they can be designed to target specific cell receptors, avoiding undesired side effects. Furthermore, they present advantages over other targeting agents (i.e., antibodies), such as their small size, which enhances tumor penetration, their higher stability in cyclization or in D-amino acid forms, and easy synthesis protocols for their manufacture and modification. Novel peptide-based targeting agents have been constantly discovered, especially by the phage-display technique. Nevertheless, the precision and the efficiency of these delivery systems can still be further improved [82].

Small molecules

Small molecules are one of the most powerful targeting moieties due to their size, stability, reproducibility, and that they offer high ligand densities as corona on the theranostic nanomaterials. These include glycoproteins, such as gum arabic and folic acid (folate), with the latter exhibiting high binding affinity (K_d_ = 10^−9^ M) to folate receptors (α or β) overexpressed in various tumors, such as triple-negative breast, which has limited therapeutic options and poor prognosis [83]. Carbohydrate ligands also make part of small targeting moieties (i.e., dextran, galactose, glucose, mannose and their derivatives) that can selectively target and recognize lectin receptors, all affording for specific targeted delivery [81].

**Table 3:** Major overexpressed receptors on breast, prostate, and lung cancer as targets for theranostic targeting.

Membrane Receptors	Ligands	Cancer	Ref.
Hormone Receptor-Positive (80%): Estrogen receptor positive (ER+) or progesterone receptor positive (PR+)	21-[^18^F]fluorofuranylnorprogesterone (FFNP)	Breast cancer	Dehdashti et al. [84]
Human epidermal growth factor receptor-2 (HER2) (20%)	Herceptin antibody		
Gastrin-releasing peptide (GRP) (65–75% and > 90%)	Series of Bombesin (BBN) peptide conjugates	Breast, prostate, and lung cancer	Kübler and Albrecht [85], Baratto et al. [86], Tangthong et al. [87]
Somatostatin (sst_2_) > 90% (antagonist†)	Octreotide, fc[CFwKTC]T(ol) RC-121 (D- Phe-Cys-Tyr-D-Trp-Lys-Val-Cys-Thr-NH_2_)	Breast, prostate, and lung cancer	Chatzisideri et al. [88], Mukherjee et al. [89]
Triple-Negative (10–20%)—BRCA1 and folate receptors	Folate	Breast, prostate, and lung cancer	Marko et al. [90], Thakur and Kutty [91]
Prostate-specific membrane antigen (PSMA) and androgen receptor	PSMA peptide Monoclonal antibody RM2	Prostate cancer	Baratto et al. [86], Cifuentes-Rius et al. [92]
Epidermal growth factor receptors (EGFRs)	EGF, EGF-like ligands, TGF-α, and HRGs	Breast and prostate cancer	Maennling et al. [93]
Lectin-binding glycoproteins (e.g., P-glycoprotein)	Lectin	Breast cancer	Zhuo et al. [94]
Prostate stem cell antigen (PSCA)	PSCA-specific chimeric antigen receptor (CAR)-engineered T cells	Prostate cancer	Lee et al. [80]
Integrin α_v_β_3_	Various types of arginine-glycine-aspartic acid (RGD) such as c(RGDyK), c(RGDfK) and (c(RGDf[N-Me]V to target tumor-associated endothelial cells	Breast and prostate cancer	Chatzisideri et al. [88], Li et al. [95]
Transferrin receptor and urokinase-type plasminogen activator receptor (uPAR)	Vitronectin	Lung cancer	Montuori et al. [96]

Targeted Theranostic in Breast Cancer

A study by Lee et al. [97] evaluated HER2-targeted ^64^Cu-labeled nanoparticle, ^64^Cu-MM-302 (^64^Cu-labeled HER2-targeted PEGylated liposomal doxorubicin) in nineteen patients with HER2-positive metastatic breast cancer for optimal doxorubicin delivery. Results revealed that ^64^Cu-MM-302 tumor accumulation at 24–48 h post-IV infusion varied 35-fold (0.52 to 18.5% ID/kg), including deposition in bone and brain lesions due to the nature of the metastatic tumor associated with more favorable treatment outcomes attributed by increased tumor delivery, was observed with increasing dose (1–13 μg doxorubicin per gram of tumor tissue (μg DOX/g)). Liu et al. [98] developed a superparamagnetic MXene-based theranostic nanoplatform composed of tantalum carbide (Ta_4_C_3_) MXenes nanosheets doped with IONPs stabilized with soybean phospholipids (Ta_4_C_3_-IONP-SPs) nanotheranostic for targeted PTT of breast tumor in a mice model facilitated by a magnetic-field with MRI and CT imaging capabilities.

Targeted Theranostic in Prostate Cancer

Prostate-specific membrane antigen (PSMA) targeting serves as one of the major promising targets for paradigm-changing practice for improving prostate cancer patient outcomes [89]. Functionalized targeted aptamer-conjugated polymeric nanoparticles (docetaxel-PLGA/PEG-A10 aptamer) have been reported to bind to PSMA [99]. Mangadlao and colleagues [61] developed a theranostic agent composed of PSMA-1 targeted pegylated AuNPs loaded with phthalocyanine-based Pc4 (AuNP-5kPEG-PSMA-1-Pc4) for enhanced PDT. The results showed that AuNP-5kPEG-PSMA-1-Pc4 demonstrated an enhanced binding affinity (IC_50_ = 0.17 nM), this was found to be attributed by the multivalency of AuNPs, a 12-fold increase binding avidity compared to PSMA-1 alone against PSMA expressing prostate tumors; moreover, it was found that internalization was via clathrin-mediated endocytosis [100].

Additionally, this resulted in the efficient delivery of Pc4 and PDT ablation at 300 J/cm^2^ with remission 14 days post-treatment. Katti et al. [101] demonstrated the use of Mangiferin conjugated radioactive gold nanoparticles (MGF-^198^AuNPs), gum arabic ^198^AuNPs (GA-^198^AuNPs), and EGCG-^198^AuNPs for targeted delivery to prostate tumors through glucose-moiety of MGF, which led to efficient endocytosis, EPR effect of the GA, and laminin receptor specificity of EGCG overexpressed on prostate tumor cells, all collectively augmenting tumor uptake and retention; of which, ^198^Au (β_max_ = 0.96 MeV; half-life of 2.7 days) β energy emission and half-life contribute for the destruction of tumor cells/tissue. Hosoya et al. [102] developed and evaluated a theranostic targeted heat sensitive-based liposome (HSL)-containing hydrogel-based nanoplatform composed of targeting both breast and prostate tumor with a NIR irradiation facilitating photon-to-heat conversion for triggered drug delivery and multimodal imaging.

Targeted Theranostic in Lung Cancer

Lung cancer is categorized into two major subtypes, which include small cell lung cancer (SCLC) and non-small cell lung cancer (NSCLC), where NSCLC is responsible for ≥ 90% of deaths. Gamal-Eldeen et al. [103] utilized gum arabic conjugated gold nanoparticles (GA-AuNPs) for the targeted delivery of A549 tumor-bearing mice; results showed that GA-AuNPs treated lung tumor-bearing mice followed by laser exposure enhanced the antitumoral efficacy. In another study, Li et al. [104] developed and evaluated ultra-pH-sensitive indocyanine green (ICG)-pegylated polymer nanoparticles (PEG-*b*-(PR-*r*-ICG)) for fluorescence imaging-guided targeted delivery via EPR effect to A549 tumor-bearing mice for enhanced PTT.

The results revealed that PEG-*b*-(PR-*r*-ICG), when irradiated with 808 nm NIR, produced maximum thermal (T_max_) heat of 51.9–53.1 °C, which completely eliminated the tumor. Zhao et al. [105] used an *sgc8c* aptamer-functionalized Fe_3_O_4_@carbon@doxorubicin NP (Apt-Fe_3_O_4_@C@DOX), where the *sgc8c* aptamer served as an active tumor-targeting ligand by endocytosis, Fe_3_O_4_ allowed for MR imaging and PTT with a T_max_ of 55.6 °C for the optimal delivery of DOX, as a synergetic enabled nanotheranostic for monitoring of the therapeutic responses of tumor [106]. Moreover, work by Gong and colleagues [107] developed a macrophage-cancer cell hybrid membrane-coated doxorubicin (Dox)-loaded poly (lactic-co-glycolic acid) (PLGA) nanoparticle (DPLGA@[RAW-4T1] NPs) for targeting and treating breast cancer-derived lung metastases. Their results demonstrated that DPLGA@[RAW-4T1] NPs (163 ± 9.61 nm) targeted α_4_β_1_ integrin via caveolar- and Na^+^/H^+^ exchange (micropinocytosis)-mediated pathways and exhibited 88.9% anti-metastasis efficacy.

## 4. Mechanisms of Diagnosis for Breast, Lung and Prostate Cancer

The current diagnostic trend consists of non-invasive real-time imaging techniques, which often decrease systemic toxicity and discomfort to patients. Such techniques can even be extrapolated to therapy when prodrugs are administered, as the conversion of the prodrug into the active drug can be followed in real-time with imaging strategies. The use of theranostic nanoparticles as tools for imaging the drug accumulation in tumors and observing the therapeutic effects in real-time has the potential to revolutionize personalized medicine, as it would enable a more accurate prediction of the treatment outcomes and precise adjustments that might be needed to increase the therapeutic success [108].

In this regard, Chen and collaborators developed a complex nanosystem composed of three covalently linked cores (a therapeutic prodrug-activating cytosine deaminase (bCD), poly-L-lysine labeled with the fluorescent dye Cy5.5 as an imaging reporter, and a vector for siRNA delivery and targeting with Indium-111-Dotatate (or [^111^In]DOTA) for the acquisition of SPECT images). The system was functionalized with a urea-based PSMA-targeting group conjugated with maleimide-PEG-NH_2_; therefore, the nanoparticles were developed as a theranostic approach for prostate cancer. A total of 48 h after the injections, the authors observed an increased accumulation of the nanoparticles in the prostate tumors; however, more toxicity and immunogenicity studies should be performed before a clinical application could be implemented [109].

Iron nanoparticles present excellent results as contrast agents for magnetic resonance imaging, especially due to their magnetic properties. In this regard, Zhu et al. [110] created superparamagnetic iron oxide nanoparticles coupled with PSMA for targeting the nanoparticles to prostate cancer foci. The authors observed a specific uptake of the nanoparticles in PSMA-expressing cells with a significantly enhanced MRI signal.

Other excellent contrast agents for MRI are superparamagnetic iron oxide nanoparticles (SPIONs). They can be further enhanced and applied for theranostic applications, as demonstrated by Manigandan and collaborators. In their study, a self-assembled amphipathic chitosan micelle complexed with a SPION system was developed for encapsulating doxorubicin in order to treat breast cancer. The targeting strategy was the conjugation with specific anti-integrin monoclonal antibodies since those integrins tend to be overexpressed in tumors, especially metastatic ones. The tumor accumulation could be observed by MRI, and it was shown that the nanomicelle-SPION complex retention into the tumor led to a great therapeutic effect [111].

An excellent non-invasive treatment option for prostate cancers is called magnetic resonance-guided focused ultrasound surgery (MRgFUS), characterized as a non-invasive, real-time monitoring, and three-dimensional imaging approach that increases therapeutic success. Wang and collaborators developed SPIONs to be used in this approach, and the nanomaterials were PEGylated and functionalized with anti-EGFR monoclonal antibodies. The specific delivery could be followed by MRI in lung cancer in vivo, and the authors observed an increased MRI sensitivity when the SPIONs were used; therefore, they could be a useful tool for MRgFUS for a rapidly-spreading cancer, such as lung tumors [112].

Gadolinium, on the other hand, has been commonly applied as a contrast for magnetic resonance imaging due to its paramagnetic properties, its ability to shorten the T1 relaxation time, and the possibility to cross the blood–brain barrier after some kind of damage. Manganese is another metal that can be used for this purpose, and different Mn-based nanomaterials have already been developed and tested, i.e., carbon-based Mn nanoparticles for brain imaging and composite Mn-Au nanoparticles as MRI contrast for stem cell labeling [113].

Dufort and co-workers developed rigid nanoparticles composed of gadolinium-based polysiloxane functionalized with chelating agents, such as DOTA, as theranostic agents for lung cancer. The nanoparticles exhibit optimal imaging properties for magnetic resonance imaging (MRI), SPECT, CT, and fluorescence imaging, either after intravenous or intrapulmonary administration [114], especially because gadolinium-based contrast agents present interesting characteristics for imaging (e.g., high paramagnetic profile due to the configuration of seven unpaired electron spins) [108].

Gadolinium was also used encapsulated in fullerenes, more specifically C82, as a magnetic contrast for MRI, providing an in vivo relaxivity 12 times higher than commercial gadolinium diethylenetriaminepentaacetic acid ([Gd]-DTPA) contrast agent. This relaxivity enhancement is probably due to the electronic interactions between the paramagnetic gadolinium-fullerene cage with water molecules surrounding the fullerenes [115]. One must bear in mind, though, that fullerenes lack water solubility unless some chemical modifications are made to the carbon cage, and they easily aggregate in an aqueous environment, such as plasma. The aggregation degree also influences their overall toxicity. Thus, the use of fullerenes for biomedical applications often requires attention and adjustments [116].

Gold nanoparticles have already been tested as contrast agents for computer tomography (CT) scans and demonstrated promising results [117]. The reasons for considering AuNPs as contrast agents are the significant electron density of gold and its surface plasmon resonance, which are optimal for CT, Raman spectroscopy, photoacoustic image, and multiphoton microscopy [118].

Compared to iodine, a common contrast for CT, gold can be thrice as good due to its superior absorption coefficient relative to all other biological tissues and tumors. By functionalizing AuNPs with targeting agents, e.g., anti-EGFR antibodies, the tumor-specificity and contrast efficiency can be significantly enhanced, as demonstrated by [119] for head and neck squamous cell carcinoma in mice.

Butterworth and colleagues tested potential theranostic AuNPs functionalized with DTPA for prostate cancer radiotherapy and found that the rectal and bladder toxicity could be significantly decreased, and the good CT contrast provided by the nanoparticles might be useful for minimizing the risk of damaging healthy tissues during radiotherapy [118].

Gold was used in the synthesis of mesoporous magnetic gold nanoclusters for drug delivery and chemo-photothermal co-therapy, while the magnetic property allows selective accumulation by extra-magnetic field and enables the use of the device as an MRI contrast agent [120].

A bimodal approach with gold and iron nanoparticles was investigated by Revia and co-workers. FeNPs could be used as contrast agents for MRI, while AuNPs are useful for X-ray computed tomography and photoacoustic imaging. At the same time, the metal nanoparticles could serve as carriers for RNA oligomers as an iRNA-based therapeutic approach; therefore, a complete theranostic system could be created and applied [121].

Another common theranostic approach consists of encapsulating a therapeutic agent in the core of a nanoparticle and functionalizing the surface of this NP with a targeting and signaling agent. Cano-Cortes et al. (2020) used polystyrene nanoparticles to encapsulate doxorubicin as a therapeutic agent, near-infrared cyanine dye (C7), and a targeting peptide (CRGDK sequence), which can be recognized by the neuropilin-1 receptor, overexpressed in triple-negative breast cancer. Polystyrene was used for the in vitro and in vivo tests due to the biostability and compatibility with the synthetic, functionalizing, and encapsulating protocols. The authors observed that the near-infrared dye provided a good fluorescence imaging of the targeted tumor with a high signal-to-noise ratio, and the tumor volume was significantly decreased due to doxorubicin [122].

While paclitaxel is one of the most used chemotherapeutics for several cancers, indocyanine green (ICG) is an FDA-approved optical contrast for diagnostic imaging. A combination of both agents with the aid of nanotechnology could provide a synergistic effect for cancer therapeutics. In this regard, Liu and collaborators took advantage of the red emission of 8 nm albumin-coated gold nanoclusters and the easy drug conjugation enabled by albumin itself and developed BSA-gold nanoclusters functionalized with hyaluronic acid, loading the material with indocyanine green (photothermal ablative agent) as a breast cancer ablative approach. The authors observed a remarkable antitumor effect, and the drug accumulation could be observed as the tumor hyaluronidase degraded the hyaluronic acid from the nanoparticles; therefore, the nanomaterials were considered promising for future anticancer theranostics [123].

Flores and collaborators reported the use of polymeric nanoparticles conjugated with folate groups for prostate cancer targeted theranostics. It was possible to follow the accumulation of the nanoparticles in the tumor tissue due to a fluorescent probe coupled to folate, responsible for targeting PSMA-expressing cells [124].

Regarding lung cancer diagnostics, nuclear imaging techniques offer the advantage of providing high-sensitivity three-dimensional images, compared to other diagnostic tools, such as chest radiography, needle biopsy, sputum cytology, bronchoscopy [125].

Quantum dots are known for their intense fluorescence generation, which could be useful for diagnostic and theranostic purposes; however, the heavy metals in their composition may cause significant toxicity; therefore, other strategies have to be used for a safe imaging protocol. Silica-based Cornell dots (C-dots) are examples in this regard, as they are composed of a silica core with embedded fluorophores and are surrounded by a PEGylated silica shell labeled with monoclonal antibodies that guide the nanoparticles to the target site. The diagnosis, in this case, is obtained by illuminating the tumor site with a near-infrared light source, which will indicate the presence of the dots bound to the tumor. This property can be used in surgical optical guidance as well [108].

Wang et al. developed NIR fluorescent InP/ZnS quantum dots functionalized with amphiphilic copolymer polylactide-b-poly (ethylene glycol) (PLA-PEG) and anti-EGFR monoclonal antibodies, resulting in theranostic micelles for EGFR-overexpressing cancers, especially triple-negative breast cancer. The fluorescence would be used for tracking and diagnosis, while aminoflavone was used as the anticancer drug. The authors observed a significant tumor volume decrease and a reduction in the drug dose needed for inhibiting tumor growth [126].

Biotinylated graphene quantum dots might be an alternative for cancer theranostics, as tumors cells tend to overexpress biotin. Graphene quantum dots, besides the lower toxicity compared to heavy metal quantum dots, exhibit optimal surface tenability properties that allow custom modifications and thus can be designed for a plethora of theranostic applications [127].

Graphene quantum dots are other non-toxic alternatives for theranostic approaches, i.e., molecular imaging, drug delivery, and photothermal therapy. Ko et al. (2017) loaded graphene quantum dots with HER and doxorubicin for a targeted approach against breast cancer. The HER-driven accumulation of the nanomaterial could be followed by the blue light emitted by the quantum dots, and the selective doxorubicin release contributed to significant inhibition of the tumor cells [128].

Another theranostic use for carbon-based dots was reported by Wu and co-workers in 2016, in a study describing carbon dots functionalized with positively-charged polyethyleneimine (PEI) and negatively-charged siRNA molecules as well as folate as the tumor-targeting moiety. The accumulation of the nanomaterials could be followed by bioluminescence (the dots absorb in 360 nm and emit in 400 nm), and the reduction of lung tumor volume in vivo could be clearly observed by the authors [129].

When it comes to radionuclide-based imaging techniques, i.e., single-photon emission computer tomography (SPECT) and positron-emission tomography (PET), nanotechnology can be very useful due to the possibility of fabricating nanomaterials labeled with for most promising theranostic radioactive agents [108].

Lutecium (^177^Lu) has been consolidated as a therapeutic radionuclide in the clinic, but other beta emitters have been calling the attention of physicians and researchers, e.g., ^67^Cu, ^47^Sc, and ^161^Tb, as they present biologically viable half-lives (2.58, 3.35, and 6.91 days, respectively), and provide theranostic possibilities, with PET and SPECT imaging potential. By combining those radionuclides with targeting ligands, such as PSMA for prostate cancer, in a nanosized carrier, the theranostic potential is considerable [130].

Simple liposomes loaded with doxorubicin, functionalized with PSMA, and radiolabeled with ^99m^Tc were synthesized by Yari and colleagues so that the accumulation and drug delivery by the liposomes could be monitored in real-time by a gamma camera in LNCaP prostate tumor cells. The results indicate that these nanomaterials might be useful for theranostic applications in solid tumors [131].

Theranostic nanodevices offering PSMA-targeting for prostate cancer can be helpful, especially if coupled with beta-, alpha-, or Auger-emitting radioisotopes for therapy and positron-emitters for diagnostics. This approach enhances the prognosis even for chemotherapeutic-resistant and metastatic tumors. ^68^Ga, for instance, shows superior PET/CT contrast properties than other isotopes, such as ^18^F, especially regarding the detection of metastases, and it has the advantage of being eluted from a ^68^Ge/^68^Ga generator; therefore, it is more available than cyclotron-derived radionuclides [132]. Virgolini and collaborators show the most recurrent isotopes used in association with PSMA for prostate cancer.

Another promising imaging modality is called photoacoustic imaging, consisting of irradiating a specific target with a near-infrared light source in the presence of targeted nanoparticles, which create acoustic pressure waves after absorbing the energy, and those waves can be detected by an ultrasound transducer. In the case of pulsed radiofrequencies being used, the imaging is called thermoacoustic instead. Both techniques have been tested with Prussian blue nanocubes coated with silica, modified with PEG, and loaded with doxorubicin, especially for breast cancer, with good results. These biophotonic technologies can improve diagnostics by enabling simultaneous multiplex imaging with different color emitters according to different targets [108].

Photodynamic therapy (PDT) can be used in theranostic approaches, as demonstrated by Mangadlao (2018). The study describes a nanoparticle system to carry a photosensitizer, Pc4, and target prostate tumors in vivo. The animals showed tumor remission after 14 days of the photodynamic treatment, and the accumulation of the nanomaterial could be observed via the dye fluorescence; therefore, this nanomaterial could be used for surgical guidance and as a theranostic agent [61].

The combination of MRI and near-infrared fluorescence for imaging-guided photodynamic therapy was the approach recently chosen by Wang and collaborators, using cathepsin-B-activatable ultrasmall superparamagnetic iron oxide nanoprobes. These probes were functionalized with a fibronectin-targeting peptide (CREKA) and the fluorescent dye squarain. The authors observed via fluorescence a selective accumulation of the probes in triple-negative breast cancer cells, and the squarain fluorescence could act as a photosensitizer for NIR photodynamic therapy [133].

## 5. Mechanisms of Treatment for Breast, Lung and Prostate Cancer

The protocol for lung cancer treatment will depend on the malignancy and stage at the time of diagnosis and may involve surgery, chemotherapy, immunotherapy, and radiotherapy. Notwithstanding, most cases are detected at advanced stages because of the difficulty of early-stage diagnosis. In these cases, frequent local tumor invasion or distant metastasis makes them not suitable for surgical intervention. Thus, systemic chemotherapy is the main treatment protocol in the seek for extending survival. The first-line chemotherapy for lung cancer consists of platinum-based drugs, such as cisplatin and carboplatin. Albeit these drugs present dose-limiting side effects, such as nephro- and cardiotoxicity, anemia, intestinal injury, peripheral neuropathy, nausea, and fatigue. Because of their high toxicity, platinum-based drugs are usually combined with other anticancer agents, such as taxanes (paclitaxel, docetaxel) or gemcitabine [134]. That way, it is critical that the development of new and more efficient therapeutics for lung cancer as current treatments present poor response and low survival rates. In this field, hybrid nanoparticles with genes, drugs, and other biomolecules have attracted attention as novel therapeutic possibilities [135].

Concerning breast cancer treatment, the first choice is to avoid mastectomy. However, tumor recurrence, even after radiotherapy, usually leaves no option but surgery. In some cases, hormonal therapy is a good option, but in cases of triple-negative breast cancer—the most complex and aggressive type of breast cancer—it is not suitable due to the absence of expression of estrogen receptors, progesterone receptors, and human epidermal growth factor receptor 2 on tumor cells. Thus, in these cases, systemic chemotherapy with drugs, such as anthracyclins and taxanes is usually the choice. Repeated chemo cycles can kill cancer cells, but often cause damage to healthy cells surrounding the tumor as well [91]. The treatment and early detection facilitated by nanomaterials can elevate the survival rate and quality of life of the patients [136]. Theranostics is a critical factor because it provides dynamic feedback through the treatment process and helps the optimization of the therapeutic operation [137].

Prostate cancer is clinically treated with systemic chemotherapy, surgery, and radiotherapy. Paclitaxel, doxorubicin, and docetaxel are commonly used drugs that, despite being able to prolong survival, their therapeutical efficacy is considered low. Moreover, they can induce several side effects, such as hair loss, nausea, cardiac, liver, and kidney toxicity and cause damage to healthy cells close to the tumors [138].

In this topic, we will briefly talk about the mechanisms of treatment concerning nanomaterials that have been being developed to improve cancer therapy.

Chemotherapy

Chemotherapy is a treatment that has been used for several types of cancer but still holds some concerns because of its lack of specificity and consequent toxicity that leads to several side effects. In that scenario, these anticancer drugs have been conjugated to nanomaterials to increase their efficiency, reducing side effects. Currently, there are some FDA-approved nanoparticle-based chemotherapeutics drugs, such as Abraxane^®®^, which is based on albumin nanoparticles loaded with paclitaxel, and Doxil^®®^ that consists of PEG nanoparticles containing doxorubicin [139].

Nanoemulsions are an alternative for conventional delivery vehicles, presenting better stability and efficacy for chemotherapy. They can be administered by different routes. The administration of paclitaxel in a nanoemulsion system has demonstrated enhanced release, permeation, and improved cellular uptake in breast cancer cells. Additionally, this type of delivery platform can provide longer retention of the drug in systemic circulation, better oral bioavailability, and safety [140,141]. Another study demonstrated the faster onset action of intravenous administration of a nanoemulsion containing DHA-SBT-1214 (omega-3 fatty acid-conjugated taxoid prodrug) to treat prostate cancer in mice. An 88% decrease in tumor growth was observed, and the nanoemulsion formulation was demonstrated to be much more efficient than Abraxane^®®^ [141,142].

Immunotherapy

Immunotherapy is a technique that stimulates the body’s immune system to kill cancer cells. These therapies may consist of antibodies with the capacity to block suppressive immune-check-points pathways, cellular therapies with dendritic cells or engineered T cells, or even vaccines that will trigger an antigen-specific immune response in cancer [141].

The latest trends in immunotherapy for breast cancer include immune checkpoint antagonists (monoclonal antibodies) that are specific for CTLA-4, PD-1, and PD-L1. Good results have been achieved with commercially available antibodies, i.e., avelumab, atezolizumab, and pembrolizumab, although some are not yet FDA-approved. Trastuzumab is an HER-2-targeting monoclonal antibody that is often used in association with chemotherapy for breast cancer ablation, prolonging the overall survival in advanced disease stages and decreasing the chances of tumor relapse in early stages [142]. Checkpoint antagonists, such as nivolumab (FDA-approved for non-small cell lung cancer), pembrolizumab, and atezolizumab, are also useful for lung cancers. Novel treatment modalities are currently in development, which associate checkpoint inhibitors, such as ipilimumab and radiotherapy, in order to increase the antitumor efficacy of those treatments [143].

As for prostate cancer, apart from the previously mentioned immune checkpoint inhibitors, some other passive therapeutic protocols are in development, i.e., chimeric antigen receptor (CAR)-T cell therapy, radiolabeled monoclonal antibodies against PSMA, and prostate stem cell antigen (PSCA) as an immunotherapeutic agent. Active therapeutic protocols include Sipuleucel-T (Provenge, FDA-approved) immunotherapy targeting prostatic acid phosphatase and some viral vectors carrying modified prostatic antigens and co-stimulatory molecules [144].

Immunotherapy is a great challenge in nanomedicine because it is paramount to guarantee the targeted delivery of immunomodulatory and immunostimulatory molecules to the correct cells; however, it holds the best promises to overcome such targeting issues [137].

Gene Therapy

RNA nanoparticles are known to present low immunogenicity and toxicity, target specificity, therapeutic component, and others very interesting properties. In addition to that, their physicochemical properties allow the delivery of siRNA and microRNA, the imaging via fluorogenic RNA and RNA aptamers, and the module for tumor targeting [145].

In gene therapy, toxic genes are delivered to cancer cells, causing their death. To do that, microRNA, an endogenously expressed non-coding RNA molecule, has been considered a target for many diseases. Further, a vector is required to guarantee the preservation of the gene in the bloodstream [137].

siRNA also holds the property of blocking or silencing the genes responsible for causing cancer. Studies have demonstrated that the presence of cholesterol ligand on the head of the arrow-shaped RNA structure induces the loading of the nanoparticles to the extracellular vesicle, allowing the delivery of siRNA to the tumor cells and blocking their growth. Researchers have reported that the delivery of siRNAs nanoparticles was able to silence prohibitin 1 expression and block the growth of prostate tumor [146,147].

Photodynamic Therapy (PDT)

Photodynamic or photochemotherapy is a treatment that involves a photosensitizing chemical substance (photosensitizer) that will be irradiated with specific wavelengths to induce the production of reactive oxygen species (ROS), for instance, singlet oxygen, peroxide, superoxide, and hydroxyl radicals. These ROS can induce cell death at the place where they are generated, and therefore, can be used as a treatment for some conditions, including cancer.

Specifically, for cancer, there is a different kind of photochemotherapy with two-photon excitation (TPE) that merges the advantages of TPE near-infrared (NIR) photosensitizers and nanotechnology [148]. As a result of the absorption of two relatively low-energy NIR photons, there will be the emission of high-energy photons in the visible spectrum that in turn will sensitize oxygen-producing cytotoxic ROS. The difference between TPE and single-photon-based PDT is the long-wavelength light used that makes it possible to reach deeper tissues, and therefore, tumors that are located deeper. Several lines of evidence show that the TPE technique may also be used for theranostics [108].

Photothermal Therapy (PTT)

Photothermal therapy presents similar principles to PDT, with the application of nanoheaters/heating agents to achieve and accumulate on the tumor site via EPR or active targeting in order to provide the localized temperature increase. This process destroys proteins and DNA/RNA molecules, inducing membrane rupture or necrosis and, thus, cell death. However, in PTT, the presence of oxygen is not necessary to kill the tumor cells. Moreover, plasmonic PTT (PPTT) has been gaining attention lately regarding the use of gold nanoparticles to be irradiated with infrared or near-infrared light, causing excitation of its conduction electrons at the surface (because of the surface plasmon resonance—SPR). When that happens, these electrons produce localized heat waves that can kill cancer cells.

Studies with nanotheranostics materials for tumor targeting, photon-to-heat conversion, and drug delivery enabling drug-controlled release for prostate and breast cancer have been described in the literature [102,107].

Therapies with radionuclides

Radionuclides applied to target therapy have been gaining attention as an option for cancer treatment for presenting the advantage of delivering high radiation doses to the tumor without compromising healthy tissues in the neighborhood.

For instance, PSMA-targeting ligands labeled with radionuclides have been used in clinical studies and indicating encouraging results for prostate cancer. The radiation used for this therapy may be of β-particles, α-particles, and Auger electrons depending on the radionuclide chosen. β-particles are emitted by radionuclides, such as yttrium-90, lutetium-177, iodine-131, and terbium-161. They have a low linear energy transfer (LET) of 0.2 Kev/µm, but enough to cause some damage to DNA, such as base chemical modifications and protein crosslinks. These particles can travel through 1–10 mm of tissue and, thus, may cause damage to surrounding cells. On the other side, α-particles have a short range in tissue, from 50–100 µm. This way, the ^4^He nucleus is suitable for small tumors, micro-metastases, and individual neoplastic cells.

However, α-particles can kill more cells with less radiation due to their capacity to induce lethal DNA double-strand breaks. Finally, Auger electrons have extremely low energy and can travel through only some nanometers. Nonetheless, radionuclides that emit Auger electrons also release γ-rays, X-rays, β-particles, and internal conversion electrons, resulting in several energy deposition distances [148].

In neutron capture therapy (NCT), radiation is generated inside the tumor site by a nuclear reaction. Usually, boron atoms are bombarded with thermal neutrons to produce α-particles—^10^B(n,α). Gd has also been considered for this type of therapy; however, the toxicity of Gd^3+^ has been a concern. Radioimmunotherapy (RIT) is a treatment based on the use of monoclonal antibodies (mAbs), or fractions of mAbs, labeled with radioactive isotopes (α, β, ou Auger-electrons emitters) to irradiate the tumor. This technique has been used for years, and the number of studies concerning nanoparticles for RIT is increasing [108].

Magnetic Therapy

The most common type of magnetic therapy uses an alternating-current magnetic field to generate hyperthermia, inducing tumor cells to apoptosis. The heat comes from the Brownian and Neel relaxation processes, and the smaller the nanoparticles used, the more the Neel relaxation processes will take over the Brownian, heating the tissue and causing cell death. There are some situations that a direct-current magnetic field is used; in that case, magnetocytolysis happens, causing cellular disruption. In this kind of therapy, magnetic nanoparticles may also be used as contrast enhancers for MRI and, therefore, work as a theranostics platform [108,149].

Iron oxide particles have been evaluated and used in magnetic resonance technology-based biomedical applications, such as multifunctional theranostic complexes, due to their magnetic properties, combining tumor targeting, imaging, and nanotherapy for personalized cancer treatment. For breast cancer, there are experiments on blocking the IL4-α receptor (IL4Rα) using PEGylated superparamagnetic iron oxide nanoparticles (SPIONs) to inhibit breast cancer cell proliferation. By blocking this receptor, there was a notable decrease in cell viability and apoptosis in 4T1 cells. Additionality, a merged treatment using SPION-IL4Rα-doxorubicin caused a significant growth in apoptosis, cell death, and oxidative stress when compared to SPION-IL4Rα or doxorubicin alone [108].

Another promising theranostic for breast cancer is pH-sensitive poly (β-thiopropionate) nanoparticles with super magnetic core and folic acid (FA) conjugation (FA-doxorubicin-iron oxide nanoparticles [FA-DOX@IONPs]) for the delivery of an antineoplastic drug, DOX, for the treatment of folate receptor (FR)-overexpressed breast cancer. Aside from their imaging function, the nanoparticles can release in response to pH 5, namely the acidic environment of the tumors. It was shown that FA-DOX@IONPs cause cellular apoptosis and have the strongest cytotoxicity against breast cancer cells when compared to free DOX or non-FR targeted nanoparticles (DOX@IONPs). Furthermore, FA-DOX@IONPs with magnetic field treatment suppressed the growth of in vivo tumors in mice much better than either treatment alone. Furthermore, the nanoparticles exerted no toxicity against other healthy organs [150].

## 6. Future Directions

The development of nanomaterials for theranostic purposes must take into consideration some difficulties that may arise regarding conjugation chemistries, synthesis, and encapsulation protocols. Theranostic materials require good compatibility among the nanoparticle core, the targeting agent, the diagnostic moiety, and the active drug [122]. The ideal nanomaterial for theranostic purposes should have a synthesis protocol with the fewest steps possible, affordable and realistic costs, high reproducibility and ease to scale-up, and should provide diagnostic and therapeutic efficiency. Targeting agents with imaging properties may improve the sensitivity and accuracy of both treatment and diagnostic outcomes, providing a real-time follow-up that is a trend in the medical field, especially in cancer treatments. In this regard, activatable prodrugs may also be applied for real-time monitoring of cancer theranostics, thus helping in the decision-making process regarding the most effective treatment approach for each patient [151].

A plethora of imaging techniques can be applied for theranostic purposes, i.e., fluorescence imaging, scintillography, tomography (PET or SPECT), magnetic resonance, ultrasound, Upconversion imaging. More than one technique may even be combined in order to increase the efficiency of the nanomaterials. Therefore, the nanomaterials for theranostic purposes must be synthesized with all those features as an ultimate goal in order to enhance the therapeutic outcomes whenever they are used.

## Figures and Tables

**Table 1:** Relevant studies using nanomaterials for breast, lung and prostate cancer and their synthesis methods.

Synthesis Protocols						
Nanopartilces	Type of Nano	Preparation Method	Applications	Type of CANCER	Source	Year
Human Serum Albumin	Organic	Desolvation technique for preparation of TRAIL/transferrin/doxorubicin HSA nanoparticles (TRAIL/Tf/DoxHSA-NPs).	Drug delivery	HCT 116, MCF-7/ADR and CAPAN-1 cell lines	Bae, S. et al. [48]	2012
Paclitaxel (PTX)-(PEG-PCL) polymer micelles	Organic	Discoidal porous silicon particles were fabricated by modification of protocols that combined electrochemical etching and photolithography. A solvent evaporation procedure was used to fabricate PTX micelles.	Chemotherapeutic; drug delivery	Breast cancer, MCF-7 and MDA-MB-468	Blanco, E. et al. [49]	2013
Maghemite NPs coated with rhodium (II) citrate	Metallic	Maghemite nanoparticles were synthesized by alkaline co-precipitation of Fe^2+^ and Fe^3+^ ions. Then Magh-Rh2Cit was prepared using 5 mL of the colloidal dispersion with 1 mL of Rh2Cit and stirred for 24 h.	Drug delivery	Bearing 4T1 breast carcinoma	Peixoto, R. et al. [50]	2015
SPIONs (MF66) (MF66-N6LDOX)	Organic/Metallic	The magnetic nanoparticles were produced by means of the co-precipitation technique and coated with oleic acid and dispersed in toluene, and a solution of DMSA in dimethyl sulfoxide (DMSO) was added to perform a ligand exchange from oleic acid to DMSA.	Drug delivery	Breast adenocarcinoma (MDA-MB-231).	Kossatz, S. et al. [51]	2015
Zn-doped TiO_2_ nanoparticles	Metallic	Titanium (IV) isopropoxide Ti [OCH (CH_3_)_2_]_4_ and zinc nitrate [Zn (NO_3_)_2_. 6 H_2_O] were prepared in ethanol and transformed into a gel prior to doping with Zn.	Cancer therapy	Breast cancer MCF-7 cells	Ahamed et al. [52]	2016
Curcumin-loaded solid nanoparticles	Organic	Empty and curcumin-loaded solid lipid nanoparticles (SLNs) were prepared by using an ethanolic precipitation technique.	Cancer therapy	Breast cancer (MCF7 and MDA-MB-231)	Minafra, L. et al. [53]	2019
Zinc Oxide	Metallic	I. Synthesis of amine-functionalized zinc oxide nanoparticles (ZnO NPs); II. Tagging of 3-carboxybenzeneboronic acid (PBA) to ZnO NPs; III. Loading of curcumin to ZnO-PBA NPs.	Drug delivery	Breast cancer	Kundu, M. et al. [54]	2019
Arginine-glycine-aspartic (RGD) tripeptide modified	Organic	Arginine-glycine-aspartic (RGD) tripeptide modified is encapsulated in pH-sensitive solid lipid nanoparticles (SLNs). RGD-HZ-GMS was applied to encapsulate doxorubicin (DOX) to construct a RGD-modified, DOX-loaded SLNs (RGD-DOX-SLNs).	Drug delivery	Breast cancer (MCF-7 and MCF7/ADR)	Zheng, G. et al. [55]	2019
Chrysin-Anchored Silver and Gold Nanoparticle-Reduced Graphene Oxide	Metallic/Non-metallic	Anticancer flavone chrysin (5,7-dihydroxyflavone ChR) was employed to fabricate silver (AgNPs), and gold nanoparticles (AuNPs) hybridized with reduced graphene oxide (rGO) nanocomposites (ChR@Ag-rGONCs and ChR@Au-rGONCs)	Cancer therapy	Breast cancer (MDA-MD-468 and MDA-MD-231)	Gnanasekar, S. et al. [56]	2020
SPIONs	Metallic	Multifunctional hybrid nanoparticles composed of iron oxide, coated with caffeic acid, and stabilized by layers of calcium phosphate and PEG-polyanion block copolymer for incorporation of siRNA that was used in magnetic delivery systems for siRNA the HER2 Gene in the case of breast cancer.	Cancer therapy	Breast cancer cell HER2-positive line HCC1954	Cristofolin, T. et al. [57]	2020
Curcumin-loaded Cellulose Nanoparticles	Organic	Cellulose curcumin (cellulose-CUR) nanoformulation was prepared in an aqueous solution in the presence of acetone with overnight stirring.	Cancer therapy	Prostate cancer (C4-2, LNCaP, DU-145; PC-3)	Yallapu, M. et al. [58]	2012
Mesoporous silica nanoparticles	Organic/Non-metallic	The MSNs (10 mg) with AgNO_3_ and BSA were prepared by an electron-deposition method.	Prostate cancer theranostic	Prostate cancer PSA detection	Wang, H. et al. [59]	2013
Curcumin-loaded PLGA/PVA/PLL nanoparticles	Organic	The curcumin-loaded organic PLGA/PVA/PLL nanoparticles were prepared by nano-precipitation technique.	Cancer therapy	Prostate cancer (PC-3; DU-145)	Yallapu, M. et al. [58]	2014
Peptide-conjugated SPIONs	Organic/Metallic	Maleimide-functionalized QDs were conjugated with targeting peptide SP204-GGGC in an aqueous solution.	Cancer theranostic	PC-3 human prostate carcinoma	Yeh, C. et al. [60]	2016
Antigen-targeted gold nanoparticles	Organic/Metallic	Pc4 loading was performed by adding 40-fold excess of Pc4 to AuNP-5kPEG-PSMA-1 solution in chloroform.	Cancer therapy	Prostate cancer, cell lines PC-3flu and PSMA-positive PC-3pip	Mangadlao, J. et al. [61]	2018
Multi-walled carbon nanotubes with PEG and anti-PSMA aptamer.	Organic/Non-metallic	To stabilize MWCNTs in a solution, PEG-coated MWCNTs were prepared, given the highly hydrophobic surface of MWCNTs. Then 50 nM AntiPSMA aptamer with 5′ modification of amino group was added to the solution and stirred for 24 h at room temperature.	Cancer theranostic	PC-3 cells overexpressing PSMA	Gu, F. et al. [62]	2018
Systemic nanoparticle-mediated delivery of PTEN mRNA	Organic	The prepare the hybrid mRNA NPs, the cationic lipid-like compound G0-C14 and poly(lactic-coglycolic acid) (PLGA) polymer coated with a lipid–PEG shell44 were used. Enhanced green fluorescent protein (EGFP) mRNA was used as a model mRNA in the presence of EGFP mRNA NP coated with ceramide–PEG.	Drug delivery	Prostate cancer; PCA cells DU145 and LNCaP	Islam, M.A. et al. [63]	2018
Gold nanoclusters as radiosensitizing agents	Organic/Metallic	To generate PSMA-targeted Au25 NCs, the ligand CY-PSMA- was combined at pH 12 with Au^3+^ ions resulting in the formation of Au25 NCs.	Cancer treatment	PC-3pip and PC3flu Prostate cancer	Luo, D. et al. [64]	2019
Serotonin conjugated IIrinotecan loaded nanomicelles	Organic	Briefly, 10 mg of TPGS and 2 mg of IRI were dissolved in 1 mL of methanol and added to 5 mL of phosphate buffer pH 4.5 under magnetic stirring and kept for solvent evaporation. For the preparation of ligand (serotonin) conjugated, targeted nanomicelles, plain TPGS was replaced with TPGS-ST conjugate and the rest of the procedure was the same as above.	Cancer chemotherapy	PC-3 human prostate cancer cells	Tunki, L. et al. [65]	2020
Hexagonal boron nitride nanoparticles	Non-metallic	hBNs were synthesized using BA as a boron source and ammonia as a nitrogen source. The synthesis was carried out in a high-temperature furnace.	Cancer therapy	Prostate cancer (DU145 and PC3)	Ciofani, M.E. et al. [66]	2020
Solid Lipid Curmcumin Nanopartciles	Organic	SLN-Curcumin(2:1), were self-assembled and combined in an O/W environment.	Cancer therapy	Non-small-cell lung cancer cell lines	Wang, W. et al.	2012
Silk Fibroin	Organic	Two methods were used to formulate silk-based particles: spray drying and spray-freeze-drying. Cisplatin was incorporated at concentrations of 0.05% (w/v) into the silk formulations. In order to produce crosslinked silk formulations, genipin was added to the silk solutions at 0.05% (w/v) prior to the incorporation of cisplatin.	Drug carrier, targeted delivery	Lung cancer cells line A549	Kim, S. et al. [67]	2015
Doxorubicin-conjugated HSA nanoparticles coated with TRAIL	Organic	Thiolated doxorubicin was conjugated with sulfo-SMCC-modified HAS in aqueous media. Dox I-NP (40 mg as I) was then suspended in 0.1 mL of TRAIL solution (1 mg/mL) and sonicated in an ice bath.	Cancer therapy	Lung cancer; H226 cell-induced metastatic tumors	Choi, S. et al. [68]	2015
Co-delivery of Doxorubicin and miR-519c Mediated by Porous PLGA Microparticle	Organic	The organic microparticles were prepared through the water-oil-water emulsion solvent evaporation method.	Drug delivery	Human lung; adenocarcinoma cell line A549	Wu, D. et al. [69]	2015
Target delivery of doxorubicin tethered with PVP stabilized gold nanoparticles	Metallic/Organic	The synthesis of AuNPs a standard reduction of HauCl4 in NaBH_4_ as a reducing and CTAB as the capping agent. AuNPs were added to PVP and conjugated with doxorubicin.	Target delivery	Human lung adenocarcinoma cells (A549), human large-cell lung carcinoma cells (H460)	Ramalingam, V. et al. [70]	2018
MDNP containing a poly(N-isopropylacrylamide)-carboxymethyl chitosan shell and (PLGA)	Organic	The PLGA core was prepared by a standard emulsion method, as previously mentioned. Briefy, 4.5 mg NU7441, 20 mg SPIO, and 90 mg PLGA (L/G ratio: 50:50, inherent viscosity: 0.15–0.25 dL/g) in 5 mL dichloromethane solution was added dropwise to 5% (*w*/*v*) PVA (MW: 13,000–23,000) solution and sonicated for 10 min at 50 W. Following overnight stirring, the solution was centrifuged at 15,000 rpm for 30 min, washed, and lyophilized to obtain the PLGA NPs.	Cancer therapy	Lung cancer cells lines A549 and H460	Menon, J. et al. [71]	2016
Folic acid (FA)-conjugated polyamidoamine dendrimer (Den)-based nanoparticle (NP) system for co-delivery of siRNA	Organic	Polyethyleneimine (PEI) was covalently conjugated to fourth-generation Poly (amidoamine) dendrimer (Den) through a biofunctionalized PEG crosslinker molecule. CDDP encapsulation into Den-PEI nanoparticles was carried out via hydrolysis. The FA-PEG-NHS was conjugated to Den-PEI-CDDP (Den-PEI-CDDP-FA) through amide covalent linkage. The siRNA was encapsulated via electrostatic interaction in Den-PEI-CDDP and DenPEI-CDDP-FA nanoparticles by mixing the nanoparticles with siRNA.	Drug delivery receptor targeted	Non-small-cell lung cancer (H1299 and A549)	Amreddy, N. et al. [72]	2017
Gold nanoparticles synthesized from Magnolia officinalis	Organic	Magnolia officinalis leaves were dilapidated to make the aqueous extract. A digestive budding method was used to separate gold nanoparticles from polyscattering nanoparticles using the digestive budding agents.	Cancer therapy	Lung cancer cells line A549	Zheng, Y. et al. [73]	2017
Synthesis of hollow maghemite (<gamma>-Fe2O3)	Metallic	The synthesis of hollow maghemite (γ-Fe_2_O_3_) particles was modified from spray pyrolysis. The particles at the exit of the furnace were collected with a permanent (Nd-Fe-B) magnet, followed by washing with DI water and ethanol and drying at 50 °C for 6 h.	Cancer therapy	Lung cancer cells line A549	Li, S. et al. [74]	2019

**Table 2:** Clinical trials using nanomaterials for several applications.

Nanoparticle	Application	Identifier
Hafnium oxide (HfO_2_) nanoparticle activated by radiotherapy	Locoregional recurrent (LRR) or recurrent and metastatic (R/M) head and neck squamous cell carcinoma (HNSCC) and lung and liver metastases from any primary cancer eligible for anti-PD-1 therapy	NCT03589339
Iron NPs. Magnetic responsive for Thermo-ablation	Prostate Cancer	NCT02033447
Superparamagnetic iron oxide nanoparticles (SPIONs) with spinning magnetic field	Osteosarcoma	NCT04316091
Magnetic nanoparticles with cultured human corneal endothelial cells	Corneal edema	NCT04894110
Carbon nanoparticles	Lymph node tracer in rectal cancer	NCT03550001 NCT04482803 NCT04759820
Liposomes containing RNA for patient-specific tumor-associated antigens and p53 RNA	Triple-negative breast cancer	NCT02316457
Nab-paclitaxel pegylated liposomal doxorubicin (PLD)	Triple-negative breast cancer or ovarian cancer	NCT03719326
Lipid nanoparticle encapsulating mRNAs encoding human OX40L, IL-23, and IL-36γ	Relapsed/refractory solid tumor malignancies or lymphoma	NCT03739931
Nab-paclitaxel/rituximab-coated nanoparticle AR160	Non-Hodgkin lymphoma	NCT03003546
Nab-paclitaxel-pegylated liposomal doxorubicin hydrochloride l	Advanced solid tumors (spread to other places in the body)	NCT03907475
Lipid nanoparticle carrying mRNA	COVID-19 vaccine	NCT04813796 NCT04860258 NCT04838847 NCT04674189 NCT04652102 NCT04515147 NCT04449276 NCT04848467
Lipid nanoparticle carrying mRNA	Respiratory syncytial virus vaccine	NCT04528719
Lipid nanoparticle carrying mRNA	Rabies vaccine	NCT03713086
Lipid nanoparticle carrying mRNA	Cytomegalovirus vaccine	NCT04232280
Lipid nanoparticle carrying mRNA	Combined human metapneumovirus and parainfluenza virus type 3 vaccine	NCT04144348
Lipid nanoparticle carrying mRNA	Advanced solid tumor malignancies	NCT03323398
Lipid nanoparticle carrying mRNA	Advanced solid tumor malignancies	NCT03739931 NCT02872025
Lipid nanoparticle carrying mRNA	Personalized cancer vaccine	NCT03313778 NCT03897881
Lipid nanoparticle carrying mRNA	KRAS vaccine	NCT03948763
Lipid nanoparticle carrying mRNA	Personalized cancer vaccine	NCT03313778 NCT03897881
Lipid nanoparticle carrying mRNA	Advanced solid tumors	NCT03946800
Lipid nanoparticle carrying mRNA	COVID-19 vaccine	NCT04821674
Size- and charge-based RNA-lipoplex nanoparticles for targeting dendritic cells	Metastatic melanoma vaccine	NCT04526899
Size- and charge-based RNA-lipoplex nanoparticles for targeting dendritic cells	Prostate cancer vaccine	NCT04382898
Size- and charge-based RNA-lipoplex nanoparticles for targeting dendritic cells	Head and neck cancer vaccine	NCT04534205
mRNA-lipoplex nanoparticles	Ovarian cancer	NCT04163094
Size- and charge-based RNA-lipoplex nanoparticles for targeting dendritic cells	Colorectal cancer, melanoma, lung cancer, bladder cancer	NCT04486378 NCT03815058 NCT03289962
Liver-targeting lipid nanoparticle	Multiple solid tumors	NCT04710043 NCT04455620 NCT04710043
Size- and charge-based RNA-lipoplex nanoparticles for targeting dendritic cells	Solid tumor	NCT04503278
Lipid-enabled and unlocked nucleomonomer agent mRNA (LUNAR^®®^)	COVID-19 vaccine	NCT04728347 NCT04668339 NCT04480957
Lipid-enabled and unlocked nucleomonomer agent mRNA (LUNAR^®®^)	Ornithine transcarbamylase deficiency	NCT04442347
Liposome	Advanced lymphoid malignancies	NCT04072458
Army liposomal formulation (adjuvant)	COVID-19 vaccine	NCT04784767
Lipid-Inorganic Nanoparticle (LION™); 15-nm superparamagnetic iron oxide	COVID-19 vaccine (repRNA)	NCT04844268
Lipid nanoparticles	Transthyretin amyloidosis	NCT04601051
Large surface area microparticles (nanoparticulates)	Urothelial carcinoma	NCT03636256 NCT04060628
Large surface area microparticles (nanoparticulates)	Pancreatic adenocarcinoma, lung cancer	NCT04314895 NCT03077685 NCT03756311
Poly(lactic-co-glycolic acid) (PLGA) nanoparticle	Esophageal Squamous Cell Carcinoma-1 positive cancers	NCT04751786
Self-assembling protein nanoparticle immunogens	COVID-19 vaccine	NCT04742738 NCT04750343
Recombinant hemagglutinin protein nanoparticle with saponin-based Matrix-M adjuvant	Influenza vaccine	NCT04120194
Recombinant spike protein nanoparticle with saponin-based Matrix-M1 adjuvant	COVID-19 vaccine	NCT04611802 NCT04368988 NCT04533399 NCT04583995

## Data Availability

No new data were created or analyzed in this study. Data sharing is not applicable to this article.
